# How to Make Loss Aversion Disappear and Reverse: Tests of the Decision by Sampling Origin of Loss Aversion

**DOI:** 10.1037/xge0000039

**Published:** 2014-12-08

**Authors:** Lukasz Walasek, Neil Stewart

**Affiliations:** 1 Department of Psychology, University of Warwick

**Keywords:** loss aversion, decision by sampling, context, prospect theory

## Abstract

One of the most robust empirical findings in the behavioral sciences is loss aversion—the finding that losses loom larger than gains. We offer a new psychological explanation of the origins of loss aversion in which loss aversion emerges from differences in the distribution of gains and losses people experience. In 4 experiments, we tested this proposition by manipulating the range of gains and losses that individuals saw during the process of eliciting their loss aversion. We were able to find loss aversion, loss neutrality, and even the reverse of loss aversion.

One of the most prolific concepts in behavioral science is loss aversion. Numerous demonstrations in laboratory experiments and field studies established that people are considerably more concerned with losses than with gains of the same magnitude. The proposition that “losses loom larger than gains” is an important property of the prospect theory, the dominant descriptive model of decision-making under conditions of risk and uncertainty (Kahneman & Tversky, 1979; Tversky & Kahneman, 1992). Loss aversion is now regarded as a stable individual difference with a specific neural representation (Rick, 2011). Loss aversion has been used to account for valuation disparities between buyers and sellers (endowment effect; Thaler, 1980), status quo bias (Samuelson & Zeckhauser, 1988), the equity premium puzzle (Benartzi & Thaler, 1995), disposition effects in finance (Weber & Camerer, 1998), or framing effects (Tversky & Kahneman, 1981), among many (see Camerer, 2005 for a review). The underlying causes of loss aversion are still not understood (Ariely, Huber, & Wertenbroch, 2005; Novemsky & Kahneman, 2005).

The validity of the loss aversion hypothesis has been criticized by several authors (Ert & Erev, 2013; Gal, 2006; Morewedge, Shu, Gilbert & Wilson, 2009; Yechiam & Hochman, 2013). For example, Gal (2006) argued that the reluctance to accept a lottery with equal chance of losing and winning the same amount of money should not be interpreted as loss aversion, but instead as a status quo bias. Indeed, if a decision to accept or reject a gamble is reframed as a choice between the gamble and zero, loss aversion is reduced (see also Ert & Erev, 2013). In another criticism, Ert and Erev (Study 3, 2013) used lists of gambles to estimate loss aversion in risky choice. By manipulating the rank position of a critical gamble, the authors were able to produce a context in which there was no evidence for absolute loss aversion.

Here, we build on these findings and offer a new psychological explanation of the origins of loss aversion. To test this account, we vary the ranges of gains and losses available across experimental conditions. In four experiments, as a direct result of this manipulation, we observe loss aversion, loss neutrality, and the reverse of loss aversion. The situations in which loss aversion occurs and reverses were predicted in advance by the decision by sampling theory (Stewart, Chater, & Brown, 2006). In decision by sampling, people are sensitive to the rank of amounts within a set sampled from memory because valuation is generated by counting favorable binary comparisons within that sample. For example, people will behave as if $12 has a subjective value of 2/5 if amounts in the sample are $5, $10, $20, $25, and $50 because $12 compares favorably in two of the five possible comparisons (Stewart, Reimers, & Harris, in press).

We will use an example from Tom, Fox, Trepel, and Poldrack (2007) because our experiments are based on their design. Tom et al. (2007) asked their participants to accept or reject various 50–50 lotteries offering a gain and a loss. Losses on offer ranged up to $20. Gains on offer ranged up to $40. This means that $10 in the range of losses has the same rank as $20 in the range of gains—both rank half way up their respective sample. Therefore, decision by sampling predicts that this asymmetry in the range of gains and losses will produce a loss aversion of 2. The top right panel in Figure 1 illustrates the ranges used by Tom et al. (2007). They found median loss aversion of 1.93, close to the decision by sampling prediction of 2. In our decision by sampling account, the results of Tom et al. (2007) are driven by the relative value of gains compared with other gains and losses compared with other losses.[Fig-anchor fig1]

We experimentally manipulated the ranges of gains and losses across participants. The other panels in Figure 1 give predictions for these conditions. Notably, if the range of gains and losses is reversed, then, following the above logic, decision by sampling predicts the opposite to loss aversion (bottom left panel of Figure 1). Finally, decision by sampling predicts that symmetrical distributions of gains and losses should produce no loss aversion (top left and bottom right panels of Figure 1). However, if the standard account of loss aversion as a stable individual difference is correct, then we should see loss aversion in all conditions.

## Experiment 1a

### Method

#### Design

**Figure 1 fig1:**
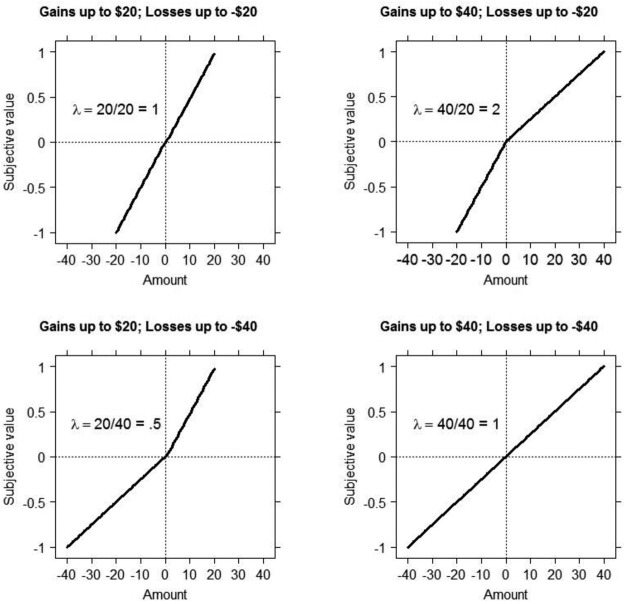
Predictions regarding the size of the loss aversion parameter under different combinations of ranges of gains and losses.

In Experiment 1a, participants were randomly allocated to one of four conditions. Both gains and losses ranged up to $20 or up to $40 generating a 2 × 2 design. We label conditions with the maximum gain followed by the maximum loss so that the 40–20 condition has gains ranging up to $40 and losses ranging up to $20. Our design creates two asymmetric conditions (20–40; 40–20) and two symmetric conditions (20–20; 40–40, as in Figure 1). Gains and losses were drawn from a list ranging from $6 to $20 (in $2 increments) or $12 to $40 (in $4 increments). Within a condition, all possible values of gains and losses were randomly paired with each other, producing 64 lotteries. Lotteries were presented in a different random order for each participant.

#### Participants

In Experiment 1a, 358 participants were recruited on Amazon Mechanical Turk (https://www.mturk.com/mturk/) and randomly allocated to one of four conditions (see online supplement). The sample size was determined in advance to give at least 95% power of detecting a medium size effect. Each person was rewarded with $0.50 for 10 min of his or her time.

#### Procedure

**Figure 2 fig2:**
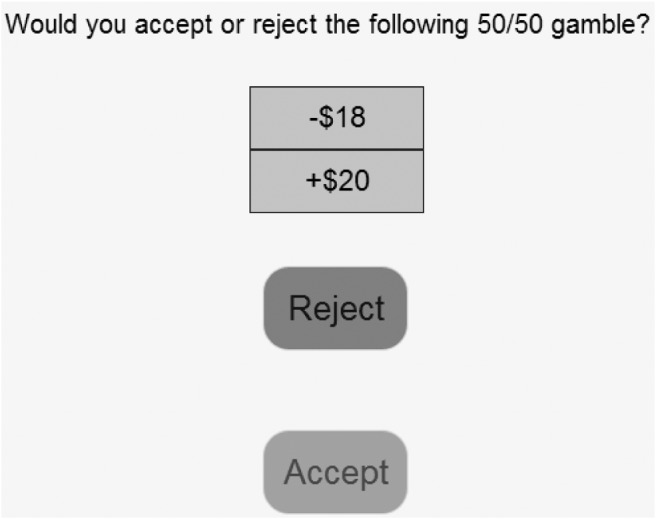
A screenshot of the main lottery task used in Experiments 1a, 1b, and 2. Experiment 3 used the same presentation format but with four buttons (Weakly Accept, Weakly Reject, Strongly Accept, Strongly Reject).

Each participant was informed that they were going to be presented with a series of hypothetical lotteries. An example lottery was displayed on the screen for illustrative purposes. Participants were told that each lottery will offer them a 50% chance of winning some amount of money and a 50% chance of losing some amount. To ensure good understanding of how the lotteries work, respondents were told to imagine an example lottery being played out by flipping a coin. The participants’ task was to simply indicate whether they would like to play a given lottery or not by pressing on accept and reject buttons (see Figure 2 for an example screen).[Fig-anchor fig2]

### Results

We estimated the loss aversion parameter for each individual by fitting a logistic regression to each participant’s responses (following Tom et al., 2007). With the assumption of linear functions for gains and losses differing only in slope (λ), and equal probability weighting for .5, the logistic regression is the same as the prospect theory model with a logit choice rule:

$$Log\left[\frac{P(accept)}{\left(1-P(accept\right)}\right]=\beta_{bias}+\;\beta_{gains}\,\;gain+\beta_{losses}\,\;loss$$ 1

In this form, the ratio of β_losses_ and β_gains_ is the loss aversion parameter λ. We include an intercept term β_bias_ to capture a general tendency to say “accept” independently of the gain and loss on offer.

We chose in advance to exclude regression fits with deviance scores in the top 5% (eight individuals). The slope for some participants was negative, which indicates the chance of accepting a lottery among these individuals decreased as their expected value increased. Because this pattern of responses shows poor understanding of the procedure, we further excluded 17 fits with a negative slope. Our exclusion criteria left us with 323 participants (90% of the sample). None of the analyses reported yields qualitatively different results if all participants’ data are retained.

Table 1 reports median loss aversion coefficients and bootstrapped 95% confidence intervals. The Condition 40–20 (top right cell) is a replication of Tom et al. (2007). In this condition, in which the range of losses is smaller than the range of gains, we replicate their standard finding of loss aversion. However, consistent with the predictions of the decision by sampling model, we find no loss aversion at all when we use symmetric ranges of gains and losses. Furthermore, when the ranges are reversed, with the range of losses being larger than that of gains, we observe that the parameter value drops below 1, signifying the opposite of loss aversion. The online Supplement reports the old-school null hypothesis significance testing (NHST) analysis for all four experiments.[Table-anchor tbl1]

## Experiment 1b

Experiment 1b is an exact replication of Experiment 1a with new participants. We decided not to run fewer participants than in Experiment 1a. A new sample of 423 participants recruited on Amazon Mechanical Turk took part in exchange for $0.50.

### Results

We used the same exclusion criteria as Experiment 1a, excluding 22 participants because of deviance scores and 19 because of negative slopes. The final sample size was 382 (90.3% of all responses).

We replicated our results in Experiment 1b (see Table 1). Once more, we found loss aversion when gains cover a wider range than losses (median λ > 1). When the ranges are reversed, we found the opposite of loss aversion (median λ < 1). In the symmetric 20–20 and 40–40 conditions, we find no loss aversion at all (median λ ≈ 1).

### Discussion

The results of the Experiments 1a and 1b are in line with the predictions of the decision by sampling model. In Experiment 2, we attempted to push the loss aversion parameter even further, using more asymmetric ranges of gains and losses. Our goal was to observe whether we can obtain even higher disparity in sensitivities to losses as a function of the values used in the elicitation task.

## Experiment 2

### Method

#### Design

In Experiment 2, we used two distributions for gains and losses, one ranging from $6 to $20 (in $2 increments) and one three times larger, ranging from $18 to $60 (in $6 increments). We only tested the two asymmetric cases. Unlike in Experiments 1a and 1b, the possible gains and losses were randomly drawn and paired from the distributions to produce 64 pairs.

#### Participants

A new sample of 429 participants recruited on Amazon Mechanical Turk took part in the experiment in exchange for $0.50.

### Results

We used the same exclusion criteria as Experiments 1a and 1b, excluding 22 participants because of deviance scores and 6 because of negative slopes. The final sample size was 401 (93.5% of all respondents).

We find a much higher loss aversion when the range of gains is higher than the range of losses. The magnitude of loss aversion parameter when the range of gains is smaller than the range of losses is not in line with our prediction, being approximately 1 rather than less than 1.

### Discussion

In Experiment 2, we found a large disparity in loss aversion estimated in different contexts. Specifically, with gains up to 20 and losses up to 60, our participants weighted losses more than twice as much as gains, whereas in the reverse condition the subjective value of gains and losses was the same. The latter finding is not in perfect alignment with our prediction of reversed loss aversion. We discuss potential reasons behind this effect in the general discussion.

The purpose of Experiment 3 was to replicate our context effect face-to-face with real incentives.

## Experiment 3

### Method

#### Design

Two ranges of gains and losses were used in Experiment 3. Monetary values could range from $5 to $20 (in $1 increments) or from $10 to $40 (in $2 increments). We chose to include both asymmetric treatment conditions, but only one of the symmetric cases (both gains and losses ranging from $10 to $40) to maximize power. Following Tom et al. (2007), every possible combination of gains and losses was used to create 256 lotteries.

#### Participants

Eighty-eight individuals from the University of Warwick participant pool were recruited for a 30-min laboratory experiment. Each person was promised to earn between £2 and £10 for their time. Our sample size was sufficient to detect a large size effect with a probability of 90%.

#### Procedure

Experiment 3 was conducted in the laboratory. At the beginning of the experimental session, each one of 88 participants was physically given £6.00 as compensation for their participation. On the computer screen, participants were shown instructions explaining the nature of the task. These instructions were identical to those used in the previous three experiments with only few notable differences. First, participants learned that they could respond using one of the four buttons, labeled as “Strongly Reject,” “Weakly Reject,” “Weakly Accept,” and “Strongly Accept” (as in Tom et al., 2007). Participants were informed that at the end of the study, one of the lotteries will be chosen at random and played out for 1/10 of the amounts, but only if that lottery was accepted. The outcome of each lottery was determined with a toss of a coin performed by the experimenter.

### Results

We used the same exclusion criteria as Experiments 1a, 1b, and 2, excluding five participants because of deviance scores and one because of negative slopes. The final sample size was 82 (93.2% of all respondents).

The results can be seen in the bottom portion of Table 1. Once again, we found that when the range of losses is smaller than the range of gains, people are more sensitive to losses. When the ranges are reversed, the parameter drops below 1 showing reverse loss aversion. When gains and losses are in the same range, people exhibit very weak aversion to losses.

## General Discussion

In four experiments, we demonstrated that loss aversion is a property of the experimental design. By simply manipulating the range of possible gains and losses, we were able to find loss aversion, loss neutrality, and even the reverse of loss aversion. These results were predicted in advance by decision by sampling. In this theory, the subjective value of a gain is derived from a series of ordinal comparisons with other gains in memory and the subjective value of a loss is derived from a series of ordinal comparisons with other losses in memory.

Some authors suggested that the aversion to mixed gambles in the accept-reject task should be largely attributed to a status quo bias (Ert & Erev, 2013; Gal, 2006) in which people are biased to reject gambles. Indeed, a reviewer suggested that our results may reflect a bias to accept half of the gambles regardless of the gains and losses. Our inclusion of an intercept in Equation 1 directly deals with a status quo bias and captures any tendency to accept or reject independent of the specific gains and losses. By including an intercept, our estimate of loss aversion, λ, is based only on differential sensitivity to gains and losses. Thus, our estimates of λ are about loss aversion and not a status quo bias. We have confirmed this by fitting simulated participants who either select half of gambles at random or select the half of gambles with the better expected value; such a tendency shows up in the intercept and not in λ. In our fits to these experiments, there are no intercept differences (see online supplement).

Complementary explanations can also help to explain boundaries of our ability to reverse the loss aversion parameter in Experiment 2. As one of our reviewers pointed out, the overly negative expected value of the lotteries presented in the condition in which the highest loss was $60 but the highest gain was only $20 might have weakened the effect of our context manipulation (Sherif, Taub & Hovland, 1958). We can only speculate what effect extreme outcomes would have on our estimates of loss aversion. It is possible that when faced with many overly negative outcomes, participants simply rejected the majority of the lotteries. It is also plausible that such extreme outcomes motivated them to pay more attention (Yechiam & Hochman, 2013) and perhaps use a different decision strategy altogether.

If our claim is that loss aversion is a property of an experiment, then how is it possible that we can observe loss-averse behavior in the field? Under our account, an asymmetry in the distribution of gains and losses in the real world, which is reflected in people’s memory, leads to loss aversion. For example, there are more small debits from bank accounts than small credits so any given value ranks more highly in the set of debits than credits, giving net loss aversion (Stewart et al., 2006). In addition, if memories of real-world and laboratory distributions mix, an asymmetry in the world should bias laboratory studies toward finding loss aversion (see online materials). This could be one reason why we still find a weak loss aversion in the symmetric condition in Experiment 3. At the same time, exposure to gains and losses can differentiate behavior of traders with different level of experience. Indeed, those with extended market expertise show weaker signs of loss aversion, represented by the tendency to undertrade (i.e., endowment effect; List, 2004). In addition, past experience of gains and losses suggests that there should be some stability in sensitivity to losses among individuals. Therefore, our account is consistent with the findings showing some parameter stability across time (Glöckner & Pachur, 2012, but see Zeisberger, Vrecko, & Langer, 2012 for evidence of poor stability).

Our findings have interesting implications for the interpretation of the functional magnetic resonance imaging data reported by Tom et al. (2007). If loss aversion is a consequence of asymmetric representation of gains and losses in memory, then the activation of various dopaminergic regions may represent just that. In other words, rather than being a locus of the asymmetric value function of prospect theory, these brain regions may simply represent relative value (see Mullett & Tunney, 2013).

## Supplementary Material

10.1037/xge0000039.supp

## Figures and Tables

**Table 1 tbl1:** Median Loss Aversion Coefficients and 95% Confidence Intervals

Experiment	Loss Range	Gain Range	
		20	40
1a	20	1.00	1.79
		[0.99; 1.01]	[1.55; 2.00]
	40	0.88	1.02
		[0.81; 1.00]	[1.00; 1.09]
		20	40
1b	20	1.01	1.59
		[1.00; 1.08]	[1.42; 1.81]
	40	0.81	1.02
		[0.75; .87]	[1.00; 1.04]
		20	60
2	20	NA	2.28
			[2.00; 2.55]
	60	1.01	NA
		[0.94; 1.07]	
		20	40
3	20	NA	1.81
			[1.45; 2.14]
	40	0.93	1.23
		[0.79; 1.00]	[1.03; 1.48]
*Note*. NA = not applicable.
