# Supplementary material for: How to Make Loss Aversion Disappear and Reverse: Tests of the Decision by Sampling Origin of Loss Aversion
Source: J Exp Psychol Gen. 2014 Dec 8;144(1):7–11. doi: 10.1037/xge0000039 (PMC4312134; doi:10.1037/xge0000039)
Supplement: Supplementary file 1 [file zfr006142598so1.docx]

**Supplemental Materials**

**How to Make Loss Aversion Disappear and Reverse: Tests of the Decision by Sampling Origin of Loss Aversion**

**by W. Lukasz & N. Stewart, 2014, *JEP: General***

**http://dx.doi.org/10.1037/xge0000039**

Details on using MTurk platform for data collection.

Growing number of social scientists now use Amazon Mechanical Turk (MTurk) as a major source of their data. The major benefit of MTurk is that it offers a large sample of online participants who complete experiments at a lower cost. Understandably, many research undertook the task to explore the quality of data that is obtained from online samples, comparing it to data gathered in the lab. The conclusions are overwhelming positive, clearly showing that MTurk is a reliable source of data (e.g. Behrend, Sharek, Meade, & Wiebe, 2011; Berinsky, Huber, & Lenz, 2012; Buhrmester, Kwang, & Gosling, 2011; Chandler, Mueller, & Ipeirotis, 2013; Paolacci, Chandler, & Ipeirotis, 2010; Stewart et al. 2014).

NHST analysis for Experiment 1a

We confirm the effects of the range of gains and losses by regressing log loss aversion on two dummy factors for the ranges of gains and losses. In our estimation, we use robust standard errors (Zeileis, 2006) due to violation of heteroskedasticity, *BP*(2) = 6.29, *p* = .043. The overall model was significant *F*(2, 320) = 29.56, *p* < .001, adjusted *R^2^* = .151. As expected, the coefficients for the *range of* gains and the *range of* losses (not to be confused with β_losses_ and β_gains_) were significant (all *p’s* < .001).

NHST analysis for Experiment 1b

Regression analysis with robust standard errors (*BP*(2) = 6.27, *p* = .044) confirms that the model is significant (*F*(2, 379) = 29.84, *p* < .001, adjusted *R^2^* = .132), and this is also true for the gain and loss coefficients (*p’s* < .001).

NHST analysis for Experiment 2

The assumption of heteroskedasticity was not violated in the Experiment 2, (*BP*(1) = .743, *p* = .389). The overall model was significant (*F*(1, 399) = 98.32, *p* < .001, adjusted *R^2^* = .196), confirming that the range of gains and losses played a role in determining sensitivity to losses among our participants (*p* < .001).

NHST analysis for Experiment 3

The assumption of heteroskedasticity was not violated in Experiment 3, *BP*(2) = .238, *p* = .888. The model is significant, *F*(2, 79) = 17.44, *p* < .001, adjusted *R^2^* = .289, confirming that the range of gains and losses played a role in determining sensitivity to losses among our participants (*p’s* < .001).

Additional analysis for the biased responding account

If participants choose to divide their choices equally between accepting and rejecting presented lotteries, we should be able to find significant differences in the values of intercept between our treatment groups. To test this account, we regressed intercept values (β_bias_) on our experimental condition independently for each experiment. In none of the analyses the differences were significant (*p*'s > .185), suggesting that biased responding was not a serious issue in our data.

Test of the order effects in our data

One could expect that this explanation makes further prediction about the effect of our manipulation on the magnitude of loss aversion at different stages of the experiment. It is possible that the influence of losses and gains stored in one's long term memory have a bigger impact early on, as it takes time for the effect of immediate context to influence one’s responses. Consequently, we should observe equal levels of loss aversion in the earliest stages of the experiment, which then diverge over the course of the experimental session. In our data, we found that the effect of our manipulation was very quick (see also Stewart, 2009; Stewart, Reimers & Harris, in press). By the time a participant completed number of trials necessary to estimate the parameter’s value; loss aversion differs as a function of the distribution of gains and losses experienced. Furthermore, values used as an example on the instructions screen present participants’ with the highest and lowest value of gains and losses for the condition to which they were assigned. This information can also offer information about the context and therefore impact responses at the earliest stage of the experiment.

We also fitted a logistic regression (Equation 1) to participants’ responses on the first and last trial, independently. The estimates for each condition in all four experiments experiment are not easy to interpret. First, no big differences were observed between the estimates for the first and last trials. Second, the bootstrapped confidence intervals revealed a considerable variability in our estimates. We therefore chose not to draw strong conclusions about the order effects from this analysis.
